# Supplementary material for: Split-Belt Training but Not Cerebellar Anodal tDCS Improves Stability Control and Reduces Risk of Fall in Patients with Multiple Sclerosis
Source: Brain Sci. 2021 Dec 31;12(1):63. doi: 10.3390/brainsci12010063 (PMC8773736; doi:10.3390/brainsci12010063)
Supplement: Supplementary file 1 [file brainsci-12-00063-s001.zip › Supplementary Table S1.pdf]

**Supplementary Table S1.** Clinical and demographic characteristics of patients with multiple sclerosis (PwMS). Continuous data are expressed as mean ± standard deviation and ordinal data as median [range].

| $PwMS_{Real}$ | Age | Gender | T25FW (s) | tDCS-mode | Duration of MS (y) | Disease Subtype | Current DMT | EDSS | $PwMS_{Sham}$ | Age | Gender | T25FW (s) | tDCS-mode | Duration of MS (y) | Disease Subtype | Current DMT | EDSS |
|---|---|---|---|---|---|---|---|---|---|---|---|---|---|---|---|---|---|
| 1 | 32 | F | 8.46 | Real | 5 | RRMS | - | 3.5 | 1 | 56 | F | 6.05 | Sham | 3 | | TR | 2 |
| 2 | 50 | M | 5.67 | Real | 9 | PPMS | FN; OR | 3 | 2 | 49 | F | 8.24 | Sham | 30 | PPMS | NAT | 3.5 |
| 3 | 43 | M | 15.01 | Real | 15 | SPMS | - | 6.5 | 3 | 39 | F | 5.12 | Sham | 8 | | DMF | 4.5 |
| 4 | 53 | M | 5.73 | Real | 23 | RRMS | IF | 3 | 4 | 36 | F | 5.46 | Sham | 16 | RRMS | OR | 3 |
| 5 | 48 | M | 6.85 | Real | 19 | RRMS | DMF | 4.5 | 5 | 44 | F | 5.67 | Sham | 12 | SPMS | FN | 3.5 |
| 6 | 51 | M | 7.19 | Real | 8 | PPMS | - | 3.5 | 6 | 35 | M | 5.87 | Sham | 4 | RRMS | | 2 |
| 7 | 31 | M | 5.47 | Real | 16 | RRMS | - | 4.5 | 7 | 61 | F | 4.04 | Sham | 1 | RRMS | | 3 |
| 8 | 63 | M | 17.09 | Real | 16 | PPMS | - | 6 | 8 | 53 | M | 9.84 | Sham | 0 | PPMS | OR | 5 |
| 9 | 59 | F | 5.67 | Real | 19 | RRMS | TR | 3 | 9 | 42 | M | 4.97 | Sham | 8 | PPMS | | 4 |
| 10 | 64 | F | 10.79 | Real | 30 | SPMS | GA | 4.5 | 10 | 54 | F | 6.67 | Sham | 18 | SPMS | FM | 5 |
| 11 | 53 | F | 5.77 | Real | 23 | RRMS | IF | 3 | | | | | | | | | |
| 12 | 51 | F | 10.80 | Real | 13 | SPMS | - | 6 | | | | | | | | | |
| **Mean ± SD** | **49.83 ± 10.46** | | **8.71 ± 3.94** | | **16.33 ± 7.10** | | | **4.0 [3-6.5]** | | **46.90 ± 9.00** | | **6.19 ± 1.70** | | **10.00 ± 9.30** | | | **3.5 [2–5]** |

Abbreviations: F = female; M = male; RRMS = relapsing-remitting multiple sclerosis; SPMS = secondary progressive multiple sclerosis; PPMS = primary progressive multiple sclerosis; EDSS = Expanded Disability Status Scale; T25FW= timed 25-foot walking test; R= right-handed, L = left-handed; MS= multiple sclerosis; DMT = disease-modifying therapy; IF = interferon beta; FN = fingolimod; GA = glatirameracetate; DMF = dimethyl fumarate; FM = fampridine; NAT = natalizumab; OR= ocrelizumab; TR = teriflunomide.
