# Supplementary figures and images for: Split-Belt Training but Not Cerebellar Anodal tDCS Improves Stability Control and Reduces Risk of Fall in Patients with Multiple Sclerosis
Source: Brain Sci. 2021 Dec 31;12(1):63. doi: 10.3390/brainsci12010063 (PMC8773736; doi:10.3390/brainsci12010063)

**Supplementary Figure S1.** Recruitment process of the study.

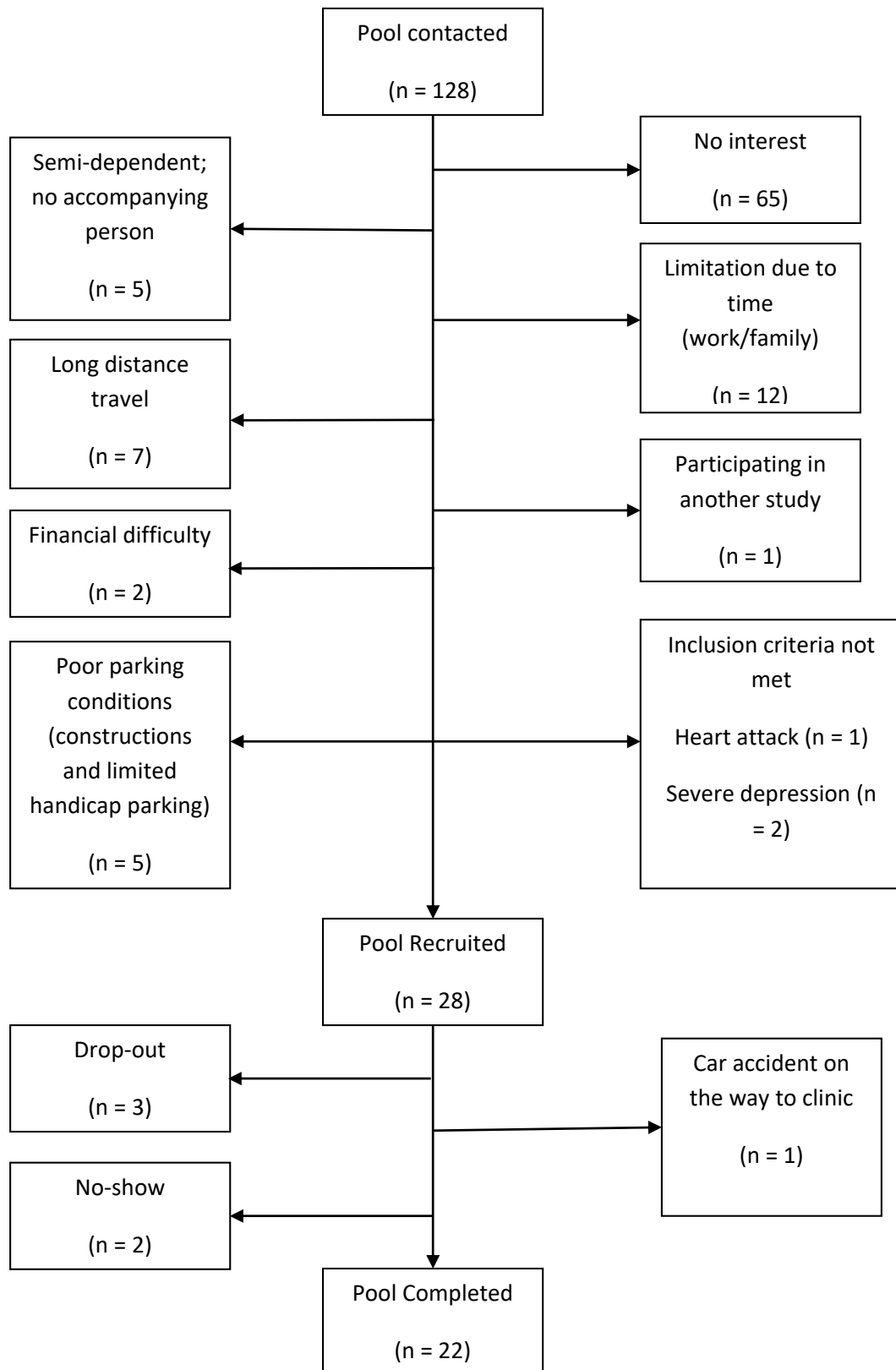

Supplement: Supplementary file 1 [file brainsci-12-00063-s001.zip › Supplementary Figure S1.pdf]
